# Editorial: *Xenopus* Models of Organogenesis and Disease

**DOI:** 10.3389/fphys.2020.00534

**Published:** 2020-05-29

**Authors:** John N. Griffin, Karen J. Liu, Emily Sempou

**Affiliations:** ^1^School of Biological Sciences, University of East Anglia, Norwich, United Kingdom; ^2^Centre for Craniofacial and Regenerative Biology, King's College London, London, United Kingdom; ^3^Pediatric Genomics Discovery Program, Departments of Pediatrics and Genetics, Yale University School of Medicine, New Haven, CT, United States

**Keywords:** *Xenopus*, development, organogenesis, disease model, oncology, patterning, regeneration

We have long extrapolated from animal models to better understand our own biology and health. Amongst such models, amphibia, and in particular *Xenopus*, have emerged as a powerhouse of biological discovery, providing startling insights into fundamental processes in embryology, cell biology, genetics, physiology, toxicology, evolution, ecology, and disease. Indeed, research in amphibians has consistently thrown open new fields of discovery, a fact reflected in contributions to numerous Nobel prizes in Physiology or Medicine, beginning with August (Lindstedt, 2014) prize for discovery of capillary motor-regulating mechanism and most recently with John Gurdon's 2012 award for reprogramming mature cells to pluripotency (Krogh, 1919; Gurdon et al., 1958; Gurdon and Hopwood, 2000; Burggren and Warburton, 2007; Blum and Ott, 2018). Over the last 70 years, *Xenopus* has emerged as the predominant amphibian model and one of most widely used model systems globally, making a tremendous impact on biological research.

Native to south and central Africa, *Xenopus laevis* initially expanded into European and North American laboratories in the 1930's and 40's as the leading pregnancy test of the time; one injection of human urine containing gonadotrophic hormone is sufficient to induce egg laying within hours (Gurdon and Hopwood, 2000). However, this ability to produce thousands of eggs and externally developing embryos on demand year-round by simple hormone injection gave *Xenopus* a distinct advantage over other available experimental models. This, combined with its large oocytes and embryos that are well-suited to biochemical, cell biological and embryological manipulations, its ease of genomic manipulation, its relative evolutionary proximity to humans, low maintenance, short life cycle, and low cost, continue to make *Xenopus* an exceptionally valuable model. In the past two decades, the establishment of *X. tropicalis*, a diploid species, as a laboratory model has added additional powerful genetic tools (Grainger, 2012; Tandon et al., 2017). Together, *X. laevis* and *X. tropicalis* allow us to rapidly investigate fundamental biological processes both *in vivo* and *ex vivo*. This makes *Xenopus* an ideal system in the genomic era, where we are in need of efficient models suitable for testing human disease gene function.

The purpose of this Research Topic is to highlight the outstanding versatility and utility of *Xenopus* as a model system in which to investigate human development, disease, and pathology. It comprises 18 primary research and review articles exploring a diverse array of topics, including development, regeneration, cancer, biological scaling, and human disease modeling, as well as providing an overview of the extensive resources available to support *Xenopus* research. It is our hope that it will be a resource both for established *Xenopus* researchers, and *Xenopus* newcomers looking to identify the appropriate model system and approach for their research.

Several articles in this Research Topic illustrate the power of *Xenopus* in modeling and investigating a broad variety of inherited human diseases. For example, Mills et al. investigate the genetic and developmental causes of Wolf-Hirschhorn Syndrome (WHS), a multigenic disorder that results in characteristic facial abnormalities. In particular, they determine requirements for four distinct WHS candidate genes during cranial neural crest migration and facial morphogenesis. Depletion of these genes in frog can disrupt facial morphogenesis, recapitulating much of the patient phenotype. Importantly, this work demonstrates the relative ease with which complex multigenic syndromes can be dissected in *Xenopus*. Expanding upon this point, Lasser et al. contribute a complementary review of WHS and discuss how *Xenopus* might be exploited to further investigate the ontogeny of this and other multigenic conditions. Ott et al. identify and functionally analyze novel compound heterozygous variants of *PIBF1* that were identified in a Joubert syndrome patient. Importantly, they discover that these disease variants affect cilia function and discuss their likely contribution to the disease. In other examples, Sempou et al. report an unexpected role for the heterotaxy candidate gene, *Fgfr4*, in gastrulation and development of the left—right body axis, providing insight into the origin of the patient phenotype, while Popov et al. use *Xenopus laevis* to determine the functional consequence of a candidate disease variant in *YWHAZ*, and investigate the molecular mechanisms underlying its contribution to the RASopathy, Cardiofaciocutaneous syndrome. Lichtig et al. develop a *Xenopus* model of Bainbridge-Ropers syndrome and reveal that depletion of the *asxl3* disease gene perturbs early neural development. In doing so they produce a powerful tool for further investigation of the condition. Finally, Hwang et al. review recent methodological advances that allow organ specific phenotypic investigations in *Xenopus* and discuss their utility in modeling genetic disease. Together, these articles add a wealth of knowledge to our understanding of congenital disease.

Technological advances are rapidly augmenting the *Xenopus* experimental repertoire and opening innovative new avenues of investigation. This is strongly evident in the field of oncology, where the marriage of classical *Xenopus* attributes and modern gene editing tools is creating efficacious new experimental platforms. In this Research Topic, Hardwick and Philpott review *Xenopus'* many contributions to our knowledge of tumor biology and discuss how genome editing technologies are revolutionizing its utility in oncology research. In addition, Dimitrakopoulou et al. highlight the untapped potential of *Xenopus* as an emerging system in which to study hematologic malignancies, and outline their experimental pipeline for generating leukemia models in *Xenopus* using CRISPR/Cas9. Deniz et al. provide another example of technological application, by demonstrating the power of hemoglobin contrast subtraction angiography as a non-destructive and efficient method to quantify cardiac function, a technique that greatly facilitates high throughput investigation of candidate congenital heart disease genes.

Several articles showcase *Xenopus'* unrivaled power for studying fundamental processes in early vertebrate development and organogenesis. For example, despite being an integral biological process, we have little understanding of how size and scaling are controlled at the cell and organism levels. Gibeaux et al. exploit the size difference between the *Xenopus* species, and the ability to generate viable intermediately sized hybrids, to investigate this mystery. Based on their findings, they propose a model whereby cell and organism size are regulated through a combination of genome size and transcriptional regulation in *Xenopus*. Haworth et al. use the ease with which embryonic tissue can be isolated and manipulated in *Xenopus* to create *ex vivo* models of cardiac and liver induction, and use these systems to explore the differential requirements for Wnt, FGF, and BMP signaling in liver formation, information critical to the refinement of protocols for liver cell differentiation from pluripotent stem cells. DeLay et al. advance our understanding of kidney development by demonstrating that the CDC42-GEF, dynamin binding protein (Dnmbp/Tuba), is essential for pronephric patterning and nephrogenesis in *Xenopus*, while Kho et al. reveal that CEP3 regulates the coordinated cell shape changes and movements required in somite segmentation.

The regenerative abilities of amphibians have long captivated biologists and inspired hope that these healing mechanisms could be applied to human injuries. While *Xenopus* tadpoles can readily regenerate damaged tissues, this ability is lost during metamorphosis, making them an ideal system for studying both the mechanisms that promote and prevent regeneration. Furthermore, as developmental processes have been extensively studied in *Xenopus*, it is an ideal model in which to examine regeneration. In this collection, Kha et al., take advantage of *Xenopus* tadpoles' ability to regrow a functional and morphologically normal eye, to investigate how similarly developmental processes are employed during embryogenesis and regrowth following injury, while Kakebeen and Wills review the biophysical, biochemical, and epigenetic processes that underlie regeneration.

*Xenopus* research is supported by powerful resources, including Xenbase, an extensive online bioinformatics and research database. Three *Xenopus* resource centers also exist to support and encourage research in *Xenopus*. In this collection, Horb et al. provide an overview of these centralized resources and the support available to both specialist and non-specialist researchers, including the availability of transgenic, inbread, and mutant animals, molecular resources, training, and experimental support. Nenni et al. add a complimentary description of advancements in Xenbase, highlighting its application to the study of disease. They also report a very fitting meta-analysis of *Xenopus* research which provides a fascinating snapshot of the breath of human diseases being investigated using *Xenopus* and the diverse experimental approaches taken by the community to understand them.

We hope that this collection of articles will be of interest to the storied *Xenopus* community as well as to clinicians and investigators working in the broader field of developmental biology and disease research.

## Author Contributions

All authors listed have made a substantial, direct and intellectual contribution to the work, and approved it for publication.

## Conflict of Interest

The authors declare that the research was conducted in the absence of any commercial or financial relationships that could be construed as a potential conflict of interest.
